# Simplified Criteria for Identification of Familial Hypercholesterolemia in Children: Application in Real Life

**DOI:** 10.3390/jcdd11040123

**Published:** 2024-04-17

**Authors:** Raffaele Buganza, Giulia Massini, Maria Donata Di Taranto, Giovanna Cardiero, Luisa de Sanctis, Ornella Guardamagna

**Affiliations:** 1 Department of Public Health and Pediatric Sciences, University of Torino, 10133 Torino, Italy; giulia.massini@unito.it (G.M.); luisa.desanctis@unito.it (L.d.S.); ornella.guardamagna@unito.it (O.G.); 2 Pediatric Endocrinology, Ospedale Infantile Regina Margherita, 10126 Torino, Italy; 3 Dipartimento di Medicina Molecolare e Biotecnologie Mediche, Università degli Studi di Napoli Federico II, 80131 Naples, Italy; mariadonata.ditaranto@unina.it (M.D.D.T.); cardiero@ceinge.unina.it (G.C.); 4 CEINGE-Biotecnologie Avanzate Franco Salvatore, 80145 Naples, Italy

**Keywords:** children, familial hypercholesterolemia, children LDL-C, FH diagnostic score

## Abstract

Background: The diagnosis of familial hypercholesterolemia (FH) in children is primarily based on main criteria including low-density lipoprotein cholesterol (LDL-C) levels, increased in the proband and relatives, and its inheritance. Two other relevant parameters are symptoms, rarely occurring in children, as rare are the FH homozygous patients, and the mutation detection of related genes. The latter allows the final diagnosis, although it is not commonly available. Moreover, the application of diagnostic scores, useful in adults, is poorly applied in children. The aim of this study was to compare the reliability of criteria here applied with different scores, apart from genetic analysis, for FH diagnosis. The latter was then confirmed by genetic analysis. Methods: n. 180 hypercholesterolemic children (age 10.2 ± 4.6 years) showing LDL-C levels ≥95th percentile (age- and sex-related), the dominant inheritance pattern of hypercholesterolemia (including LDL-C ≥95th percentile in one parent), were considered potentially affected by FH and included in the study. The molecular analysis of the LDLR, APOB and PCSK9 genes was applied to verify the diagnostic accuracy. Biochemical and family history data were also retrospectively categorized according to European Atherosclerosis Society (EAS), Simon Broome Register (SBR), Pediatric group of the Italian LIPIGEN (LIPIGEN-FH-PED) and Dutch Lipid Clinic Network (DLCN) criteria. Detailed kindred biochemical and clinical assessments were extended to three generations. The lipid profile was detected by standard laboratory kits, and gene analysis was performed by traditional sequencing or Next-Generation Sequencing (NGS). Results: Among 180 hypercholesterolemic subjects, FH suspected based on the above criteria, 164/180 had the diagnosis confirmed, showing causative mutations. The mutation detection rate (MDR) was 91.1%. The scoring criteria proposed by the EAS, SBR and LIPIGEN-FH-PED (resulting in high probable, possible-defined and probable-defined, respectively) showed high sensitivity (~90%), low specificity (~6%) and high MDR (~91%). It is noteworthy that their application, as a discriminant for the execution of the molecular investigation, would lead to a loss of 9.1%, 9.8% and 9.1%, respectively, of FH-affected patients, as confirmed by the genetic analysis. DLCN criteria, for which LDL-C cut-offs are not specific for childhood, would lead to a loss of 53% of patients with mutations. Conclusions: In the pediatric population, the combination of LDL-C ≥95th percentile in the proband and the dominant inheritance pattern of hypercholesterolemia, with LDL-C ≥95th percentile in one parent, is a simple, useful and effective diagnostic criterion, showing high MDR. This pattern is crucial for early FH diagnosis. EAS, SBR and LIPIGEN-FH-PED criteria can underestimate the real number of patients with gene mutations and cannot be considered strictly discriminant for the execution of molecular analysis.

## 1. Introduction

Heterozygous familial hypercholesterolemia (HeFH) is one of the most common genetic disorders. The estimated worldwide prevalence is about 1:300 [1] among the general population and 1:16 among subjects with coronary artery disease [2], while homozygous familial hypercholesterolemia (HoFH) is a rare and life-threatening condition [3].

Most cases of HeFH are caused by autosomal dominant mutations in the LDL receptor (LDLR) gene, although less common mutations in other genes coding for proteins involved in cholesterol metabolism or LDLR function and processing, such as Apolipoprotein B (ApoB) and (PCSK9), can be causative [4].

HeFH is characterized by abnormally elevated serum levels of low-density lipoprotein cholesterol (LDL-C). A consistent association between the magnitude and the duration of exposure of the vasculature to LDL-C and the risk of atherosclerotic cardiovascular disease was reported [5]. In childhood, a relationship between hypercholesterolemia and atherosclerotic disease in autopsies was described from two years of age [6]. In pediatric patients with HeFH, the early onset of atherosclerosis was assessed by different indicators, as ultrasound evaluation of intima media thickness (cIMT), flow-mediated dilation (FMD) of the brachial and femoral artery or with inflammatory, hemostatic and other indirect serum markers of the atherosclerotic process [7].

Different studies showed that treatment with statins from childhood can reduce total cholesterol (TC) and LDL-C burden [8], improve the endothelial function [9] and stabilize cIMT [10], then attenuate the development of atherosclerosis. Despite this evidence, FH is severely underdiagnosed (less than 1% are identified in most countries) and undertreated [11].

Since the definite diagnosis of HeFH in pediatric age is crucial, also for planning the correct treatment, efforts have been made to overcome limitations. Childhood is the optimal period to discriminate between HeFH and non-HeFH based on the LDL-C concentration, due to the minimal environmental and hormonal influences [12,13]. Further advantages are the relatively high HeFH prevalence, the availability of an easy biomarker and the generally accepted notion that the disorder is life-threatening [11]. Even so, some critical issues are still unsolved, including the lack of univocal criteria for screening. When elevated LDL-C levels are recognized, the need for a definite diagnosis is important and achievable through genetic testing.

Diagnostic criteria based on scores attributed to the family history, clinical and biochemical parameters have been made available by scientific societies including the European Atherosclerosis Society (EAS) Consensus Panel [12], Simon Broome Register (SBR) Group [14], Pediatric group of LIpid transPort disorders Italian GEnetic Network (LIPIGEN-FH-PED) [15] and Dutch Lipid Clinic Network (DLCN) [16]. EAS established three different cut-offs of LDL-C to determine a high probability of HeFH for the general population, subjects with suggestive family history and those with a parent with genetic diagnosis [12]. SBR, LIPIGEN-FH-PED and DLCN share criteria based on pre-treatment LDL-C concentrations, clinical presentation, dominant transmission and premature coronary disease in relatives. As it concerns LDL-C levels, LIPIGEN-FH-PED is specific for pediatric age, and SBR criteria specify cut-offs for subjects under 16 years. DLCN was developed for the adult population, it was not meant for use in pediatric cases and does not indicate LDL-C ranges for pediatric age. Damgaard et al., in the Danish population, evaluated for the first time the ability of different diagnostic criteria (SBR, DLCN and MEDPED) as predictors of an HeFH-causing mutation in adults [17], but, to the best of our knowledge, there are no similar studies in pediatrics.

This study had two goals: the primary was to evaluate the suitability and the results of HeFH diagnosis in children, based on revisited clinical and anamnestic features, then verified by genetic analysis. The second objective was to compare these results with those obtained by the application of the abovementioned criteria for FH diagnosis in children.

## 2. Materials and Methods

### 2.1. Patients

The study was conducted on 180 outpatients at the Lipid Clinic of Regina Margherita Children’s Hospital (Turin, Italy) in the period 2010–2019. They had been invited by pediatricians or general practitioners to perform blood lipid tests as hypercholesterolemic or at cardiovascular (CV) risk as part of a screening program. Participants included 90 males and 90 females, with an average age of 10.4 ± 4.6 years at study baseline. Children underwent two subsequent visits. On both occasions, they were subjected to clinical examination, anthropometric assessment and biochemical analysis. At the first visit, dietary counseling was also provided, and they were asked to fill in a weekly dietary diary, then evaluated by an experienced nutritionist.

The family medical history was meticulously collected. It was extended to three generations, then including the probands, their parents and grandparents, with particular attention to the relatives on the same line of the probands on the family tree. The lipid profile of the parents was required if not previously available. Attention was paid to ascertain coronary heart disease (CHD), particularly premature ones.

### 2.2. Inclusion Criteria

The inclusion criteria consisted of LDL-C ≥95th, age- and sex-related percentile (cut-off 129–140 mg/dL) [18], after 6 months of dietary compliance to Cardiovascular Health Integrated Lifestyle Diet (CHILD) 1 and CHILD 2 diets [19], each of these followed for three months; autosomal dominant inheritance of hypercholesterolemia, referred to one biological parent with LDL-C ≥95th percentile [20] (considering pre-treatment values).

### 2.3. Exclusion Criteria

Secondary hypercholesterolemia was clinically ruled out by a small array of biochemical analyses to exclude renal and liver disease, hypothyroidism, diabetes, obesity and, in general, chronic disorders requiring therapy.

Combined hyperlipidemia was ruled out with the exclusion of recognized criteria that include increased triglycerides (TG) in the proband and in family members, variation among relatives of lipid phenotype and dominant inheritance.

### 2.4. Study Design

All participants, after being submitted to the above clinical and familial investigations, and supposed to be HeFH-affected, underwent genetic analysis to reach the definite diagnosis.

Their biochemical, clinical and familial data were then retrospectively applied to EAS, SBR, LIPIGEN-FH-PED and DLCN criteria, except for genetic analysis, for the computation of scores [12,14,15,16]. They were classified into different categories (i.e., possible, probable and so on), according to the above-cited scores. The term “not applicable”, which became necessary only for EAS and SBR, was used for subjects without sufficient characteristics for categorization in the mentioned classes.

This design was finalized to compare different methods of preliminary HeFH diagnosis. The application and the reliability of these sets of criteria, score-based, were evaluated in relation to the results of genetic analysis, calculating the sensitivity, the specificity and MDR.

### 2.5. Lipid Measurement

Blood samples were obtained after overnight fasting. Total plasma cholesterol (TC), high-density lipoprotein cholesterol (HDL-C) and TG were measured with routine enzymatic methods with the Olympus 2700 Analyzer (Olympus Co Ltd., Tokyo, Japan). Plasma LDL-C was calculated according to the Friedewald formula: LDL-C = CT − (HDL-C + TG/5). LDL-C values were compared to population-based age- and sex-specific percentiles derived from the Lipid Research Clinics Program [18].

### 2.6. Genetic Analysis

For patients recruited before 2019, genetic screening was performed by a step-by-step protocol based on traditional methods, as previously described [21]. Firstly, we analyzed the LDLR gene by amplification and direct sequencing of all exons and the adjacent intronic regions. If no pathogenic variants were detected, Multiplex Ligation-dependent Probe Amplification was performed, as previously reported, to search for large rearrangements in the LDLR gene. When no pathogenic variants were identified in the LDLR gene, the sequencing of all exons and flanking intronic regions of the PCSK9 gene was performed [22,23]. In case of negativity to the genetic screening, the last step was the screening of the APOB gene, which included the region coding for the LDLR binding region, i.e., a portion of exon 26 and exon 29 together with the flanking exon–intron junctions [21]. For the other patients, genetic screening was performed by NGS using the Devyser FH v2 kit for sequence enrichment and the Illumina MiSeq for sequencing with paired-end reads (2 × 150 base pairs). This method allowed us to search for small variants in LDLR, APOB, PCSK9, LDLRAP1, APOE and STAP1 genes and for a copy number variant of the LDLR gene. Sequence analysis was performed by the Amplicon Suite software version 3.5.1 (SmartSeq), and all rare variants were evaluated for their pathogenicity according to the FH-specific suggestions to the American College of Medical Genetics and Genomics Guidelines made by ClinGen [22,24]. The data on the variants were collected consulting the Genome Aggregation Database (GnomAD) for the minor allele frequency (MAF) and the pathogenic variants databases Human Gene Mutation Database (HGMD) professional, Leiden Open Variation Database (LOVD 3.0) and ClinVar (NCBI) for previous identification of variants in FH patients.

### 2.7. Statistical Analysis

Sensitivity, specificity and mutation detection rate (MDR) were calculated for any criterion considered and compared to the results of molecular genetic analysis. Sensitivity is the ability to identify the individuals with mutations in an analyzed gene (true positive rate), i.e., the number of mutated patients identified as FH by clinical criteria divided by the total number of mutated patients in our population. Specificity is the proportion of patients without a mutation who did not fulfill the clinical criterion of FH (true negative rate), i.e., the number of patients without mutation, correctly identified as not suspected as FH-affected by clinical criteria divided by the number of patients without mutations. The mutation detection rate is the proportion of patients, all of whom fulfilled the criterion in question, in whom a mutation was identified.

## 3. Results

Among the 180 children potentially affected by FH, as a result of inclusion criteria (biochemical tests and the two generations’ data collection) and therefore submitted to molecular investigations, 164 had the final diagnosis of FH. Gene mutations were detected in the LDLR (n. 162 subjects), APOB (n.1 subject) and PCSK9 (n.1 subject) genes. The MDR was 91.1%. Clinical characteristics and lipid profiles of patients are shown in Table 1.

The mean LDL-C values, expressed in mg/dL, were 218.7, 220.1 and 203.6, respectively, in the whole participants, in the M+ (positive to gene analysis) and in the M− (negative to gene analysis) groups. In the overall I- and II-degree relatives (i.e., parents and grandparents), the percentage of CHD was 77.8%, while that of premature coronary heart disease (pCHD) was 51.1%, including 14.4% in parents. The application of different diagnostic criteria allowed us to distinguish different categories of suspected HeFH diagnosis and to consider, for each one, the number of genetically confirmed HeFH cases (Table 2). For any category of clinical suspect HeFH, the sensitivity and the specificity were calculated (Table 3).

It should be noted that all patients recruited with our criteria showed a LIPIGEN-FH-PED score ≥ 3, being then categorized as at least “possible FH”, thus making the sensitivity of 100% for this class.

As it concerns patients with a “definite FH” diagnosis according to SBR and DLCN criteria, the MDR and specificity were 100%, while the sensitivity was 4.3 and 5.5%, respectively. The LIPIGEN-FH-PED criteria included a higher number of subjects in the “definite” category, with higher sensitivity, but still only 41.5% of patients with a mutation would have been identified.

The most consistent group of probands was classified as “highly probable” for EAS, “possible” or “definite” for SBR and “probable” or “definite” for LIPIGEN-FH-PED. Their sensitivity was, respectively, 90.9%, 90.2% and 90.9%. MDR and specificity were close to 91% and 6%, respectively. Different results characterized the group “probable” or “definite” for DLCN criteria, which sensitivity was 47.0%.

The abovementioned categories include most cases addressed to the molecular analysis to reach the final diagnosis. The use of EAS, SBR, LIPIGEN-FH-PED and DLCN as discriminating to perform gene mutation detection would have resulted in a loss of 9.1%, 9.8%, 9.1% and 53.0%, respectively, of HeFH patients showing a mutation.

The inclusion of patients classified as “possible” FH, according to the LIPIGEN-FH-PED and DLCN parameters, increased the sensitivity (reaching 100% for LIPIGEN-FH-PED), but decreased the specificity, maintaining a high MDR.

## 4. Discussion

FH is severely underdiagnosed, particularly in childhood. Considering the increased CV risk, related to the exposure to high levels of cholesterol since pediatric age, it is necessary to find the easier, but most accurate, methods to reach the diagnosis. Although the gold standard is represented by molecular analysis, it should be considered as a second-level tool, due to the cost and availability. The selection of children to be addressed to genetic analysis is a relevant aspect in medical practice.

Two main criteria need consideration facing the hypothesis of HeFH in hypercholesterolemic children: the LDL-C level and the familial history, clinical symptoms being uncommon.

The LDL-C concentration in children greatly varies, but the 95th percentile is commonly considered the cut-off to classify elevated levels. In our study, we used those derived from the Lipid Research Clinics Program [18]. The Dutch Lifelines Cohort Study introduced more recently pediatric lipid references, since 8 years of age, and even applying these updated percentiles, our results are confirmed [25].

Wiegman et al. showed that LDL-C levels >3.50 mmol/L (~135 mg/dL) had a 0.98 post-test probability of predicting the presence of an LDL receptor mutation in children from families with known HeFH [26]. Concerning the family history, the parents, commonly young, infrequently experience myocardial infarction or suffer coronaropathies, as also recently described by LIPIGEN [27]. In this scenario, it is mandatory to reach the second-generation data.

In this study, we firstly assessed the final diagnosis of HeFH by gene analysis in children with LDL-C ≥95th percentile, age- and sex-related percentile (values between 129 mg/dL and 140 mg/dL [18]) and the autosomal dominant inheritance of hypercholesterolemia, considering, besides parents, also grandparents to confirm the heritage pattern and to fill the gap, at times, of cholesterol level knowledge by parents. The LDL-C ≥95th percentile was considered for parents, while other methods here considered used a single cut-off for adults, regardless of the age and potentially excluding some young adults with lower LDL-C levels [18]. This implies that our criteria are less restrictive than proposed by the above-cited strategies.

The parental lipid profile is a crucial element for the diagnosis. Williams et al., in a study conducted on adults from the general population, at a TC level of 310 mg/dL found 4% of adults HeFH-affected, while 95% of their first-degree relatives, with the same TC levels, were demonstrated to be HeFH-affected [28].

Van der Graaf et al., in 1430 children presenting LDL-C ≥95th percentile and autosomal dominant hypercholesterolemia (at least 1 biological parent on treatment for hypercholesterolemia and a family history of hypercholesterolemia and cardiovascular disease), reported an MDR of 95%, higher than previous studies [29]. This study is comparable to the present one as they share the methodology, are children-centered and underline the relevance of a rigorous familial study.

Secondly, we analyzed the application of different score sets based on clinical, biochemical and familial criteria, as proposed by EAS, SBR Group, LIPIGEN-FH-PED and DLCN to compare their sensitivity, specificity and MDR. The issue of their reliability with respect to the need for molecular genetic analysis is relevant for medical practice.

DLCN and SBR are the most used diagnostic criteria in adults. DLCN does not have a specific cut-off for pediatric age, then it has great limitations if applied to children, as our study confirmed. The diagnosis of “probable” or “definite” HeFH, as a discriminator to perform gene analysis, would have led to a loss of 53.0% of patients resulted as HeFH-affected by gene mutation detection (i.e., a sensitivity of 47%).

The molecular analysis in children is usually suggested in the case of “highly probable” FH for EAS, “possible” or “definite” for SBR and “probable” or “definite” for LIPIGEN-FH-PED. In such conditions, we found high sensitivity, high MDR and low specificity for all scores, without large differences among those.

Our study highlights that our very simple criteria, based only on LDL-C levels in children and in their parents, besides the identification of the second-generation clinical profile, make the identification of young FH very effective. The MDRs were similar to other criteria, but requiring less information. On these bases, these criteria could be applied also by pediatricians not experts on dyslipidemias.

The use of the scores, as a key for the execution of molecular investigations, would have led to us underestimating the real number of patients harboring a mutation, with a loss of 9.1%, 9.8% and 9.1% of affected patients, respectively. This percentage of “false negative” children for EAS, SBR and LIPIGEN-FH-PED, who would not be submitted to gene investigations, represents a misdiagnosis. In this latter group, LDL-C values vary between 137 and 153 mg/dL; two more patients with higher LDL-C were excluded for SBR and LIPIGEN-FH-PED due to parental values of TC and LDL-C not reaching the cut-offs of those criteria. To increase the specificity, only the “defined” criterion should be considered, but the lower sensitivity is unacceptable in the clinical setting for a first-level selection of patients.

The low specificity of the SBR, LIPIGEN-FH-PED and DLCN criteria could be explained by the fact that these criteria are also based on the presence of CHD in the patient relatives, which could be associated with different risk factors besides the hypercholesterolemia. According to our results, patients without mutations showed a higher prevalence of CHD and pCHD than patients with mutations, despite lower levels of LDL-cholesterol. This aspect highlights that the clinical suspicion of FH in children should be raised mainly based on the presence of hypercholesterolemia than on the presence of CHD in relatives. This conclusion validates the results of our previous study on pediatric patients collected in two centers, indicating that, among the family data, only hypercholesterolemia is more prevalent in patients with mutations than in patients without mutations [30]. The application of the present study criteria, in comparison to those by EAS, SBR and LIPIGEN-FH-PED, allowed to detect a greater number of children resulted positive to mutation detection, while maintaining a comparable MDR. A key aspect of the clinical criteria used to address patients to FH genetic screening is the number of vain genetic analyses performed. Our criteria led to a low number of analyses not revealing pathogenic variants, only 16/180 (8.9%).

These results showed that also children with increased, although not extremely elevated, LDL-C should have genetically based HeFH in the presence of dominant transmission of hypercholesterolemia (possibly evaluated in the three generations), independently by family history of CV event. They must not be lost.

In adults, different studies evaluated the relationship between HeFH clinical diagnosis and genetics with SBR and DLCN. Two main studies conducted in Denmark [17] and Australia [31] demonstrated high MDR and specificity while sensitivity was very low and vice versa, as in our series. Particularly, the SBR score showed sensitivity >90%, specificity ~30% and MDR 38% in the setting of an HeFH “probable” or “definite” diagnosis. The most remarkable difference from our study was the lower MDR in adult series, probably related to less rigorous inclusion criteria and to the higher prevalence of hypercholesterolemia. The latter might have been caused by unhealthy lifestyles in adults, whose impact is heavier than in childhood. Two other important differences must be considered between children and adults: first, in children, clinical signs such as tendon, tuberous xanthomas and corneal arcus are rare; moreover, in the pediatric age, it is easier to obtain the lipid profile of parents and other relatives, which is a crucial point for the diagnosis.

The role of molecular analysis in FH is still debated. It is the gold standard for the definitive diagnosis, it can facilitate genetic counseling, as the cascade screening, and it increases the adherence to treatment of individuals at very high risk of early CHD or preclinical atherosclerosis [12]. Furthermore, FH mutation carriers are at substantially increased risk for CHD compared to non-carriers with the same LDL-C levels [32]. Critical issues are cost and organization, besides the great presentation variability of HeFH, and need consideration. In Italy, the LIPIGEN study reported that more than 94–98% of mutations involved the LDLR gene [33,34] as here confirmed: only two cases of APOB and PCSK9 gene mutation were detected inside our children group.

Once the index case is diagnosed, cascade screening of families using a combined phenotypic and genotypic strategy is recommended from 5 years of age, or earlier if homozygous FH is suspected [12]. Cascade screening is the most common screening strategy for FH in several countries; it has some limitations, in particular the challenge of identifying the index patient, considering that FH is an underdiagnosed condition [11]. Furthermore, when performing cascade genetic screening involving trios (father, mother and child), the rate of non-paternity due to cuckoldry was estimated to be approximately 1%, [35] and, while it is rare, de novo mutations should also be considered [36]. Other strategies of screening have been proposed, such as selective or universal screening [19,37]. Universal screening would be the best way to identify all FH in a population, but the very high costs make it unapplicable in the clinical real life of most countries.

An early definite diagnosis is important to start the treatment. In the included population, a high percentage of CHD and pCHD in relatives was detected. Statin therapy should be started from the age of 8–10 years in HeFH-demonstrated subjects, as proven effective and well tolerated by 20 years of experience in children [38].

Thus, our proposed inclusion criteria, based on LDL-C values, familial clinical and anamnestic data, could be an accurate method to guess the diagnosis and select patients to undertake the molecular analysis.

## 5. Conclusions

Early identification of pediatric patients with HeFH is important for early management, primarily by pediatricians and GPs to reduce CV risk, but to date, recommendations for lipid screening in the pediatric field are still ambiguous, and there are no univocal criteria for the diagnosis.

The comparison of EAS, SBR and LIPIGEN-FH-PED led to the classical dilemma: MDR and specificity are high only if sensitivity is very low and vice versa. The use of these criteria as a discriminant for the execution of the molecular investigation would have underestimated the real number of patients positive to mutation detection and cannot be considered a good selection tool for the application of gene analysis.

Thus, we demonstrate that subjects with LDL-C ≥95th percentile and a clear autosomal dominant inheritance pattern of hypercholesterolemia, after excluding secondary causes of dyslipidemia, should be considered for the molecular analysis. This represents a simple method with high MDR, not chained to scores based on many clinical data, thus easy for the general practice application, which underlines the need to consider the proband not as a separate subject but within the family context.

## Figures and Tables

**Table 1:** Characteristics of index patients.

	Overall	Mutation	No Mutation
Number	180	164	16
Age (years)	10.2 ± 4.6	10.1 ± 4.7	11.0 ± 3.3
Sex (male/female)	90/90	84/80	6/10
Total cholesterol (mg/dL)	290.5 ± 49.7	291.6 ± 51.1	279.0 ± 30.1
LDL cholesterol (mg/dL)	218.7 ± 45.6	220.1 ± 46.5	203.6 ± 33.3
HDL cholesterol (mg/dL)	56.8 ± 12.5	56.5 ± 12.2	59.6 ± 15.5
Triglycerides (mg/dL)	75.9 ± 30.6	75.6 ± 31.3	78.6 ± 23.0
CHD in I-II-degree relatives (number, %)	140/180 (77.8%)	126/164 (76.8%)	15/16 (87.5%)
pCHD in I-II-degree relatives (number, %)	92/180 (51.1%)	83/164 (50.6%)	9/16 (56.2%)
pCHD in parents, n %	26/180 (14.4%)	22/164 (13.4%)	3/16 (18.7%)

**Table 2:** FH diagnosis in 180 children categorized according to EAS, SBR, LIPIGEN-FH-PED, DLCN and the present study criteria.

	HeFH Diagnosis	Total	Mutation	No Mutation
European Atherosclerosis Society (EAS)	Non-applicable	16	15	1
	Highly probable	164	149	15
Simon Broome Register (SBR)	Non-applicable	17	16	1
	Possible	156	141	15
	Definite	7	7	0
LIPIGEN-FH-PED	Unlikely	0	0	0
	Possible	16	15	1
	Probable	94	81	13
	Definite	70	68	2
Dutch Lipid Clinic Network (DLCN)	Unlikely	32	26	6
	Possible	70	61	9
	Probable	69	68	1
	Definite	9	9	0
Present study criteria		180	164	16

**Table 3:** The Mutation Detection Rate comparing different methods and the prediction of clinical score in the diagnosis of FH.

Clinical Criteria	HeFH Diagnosis	Sensitivity	Specificity	Mutation Detection Rate
European Atherosclerosis Society (EAS)	Highly probable	90.9%	6.3%	90.9%
Simon Broome Register (SBR)	Possible or definite	90.2%	6.3%	90.8%
	Definite	4.3%	100%	100%
LIPIGEN-FH-PED	Possible or probable or definite ≥ 3	100%	0%	91.1%
	Probable or definite ≥ 6	90.9%	6.3%	90.9%
	Definite > 8	41.5%	87.5%	97.1%
Dutch Lipid Clinic Network (DLCN)	Possible or probable or definite ≥ 3	84.1%	37.5%	93.2%
	Probable or definite ≥ 6	47.0%	93.8%	98.7%
	Definite > 8	5.5%	100%	100%
Present Study Criteria				91.1%

## Data Availability

The records of patients are available in our local Informatic Database Trak-Care System.
